# Editorial: Application of telehealth to diabetes care delivery and medical training: challenges and opportunities

**DOI:** 10.3389/fendo.2023.1229706

**Published:** 2023-06-16

**Authors:** Roeland J. W. Middelbeek, Matthew F. Bouchonville, Shivani Agarwal, Giulio R. Romeo

**Affiliations:** ^1^ Joslin Diabetes Center, Boston, MA, United States; ^2^ Department of Medicine, Beth Israel Deaconess Medical Center, Boston, MA, United States; ^3^ Harvard Medical School, Boston, MA, United States; ^4^ Division of Endocrinology, Diabetes and Metabolism, University of New Mexico School of Medicine, Albuquerque, NM, United States; ^5^ Department of Medicine, Albert Einstein College of Medicine, Bronx, NY, United States; ^6^ Montefiore Medical Center, Bronx, NY, United States

**Keywords:** telehealth, diabetes care, medical training, tele-education, remote glucose monitoring

This editorial reviews the articles included in the Research Topic “*Application of Telehealth to Diabetes Care Delivery and Medical Training: Challenges and Opportunities*” and their contribution to the broader landscape of telehealth-based interventions in diabetes management.

The manuscripts in this Research Topic address three main themes: 1. The benefits and limitations of telehealth to promote equity of access to diabetes care across a diversity of demographic groups. 2. the efficacy of e-health-based interventions, including the feasibility of remote glucose monitoring using commercially available platforms for people with diabetes; 3. the impact of telemedicine on medical training during the COVID-19 lockdown period.

In the setting of the expanding socioeconomic burden of health inequities (1), telehealth has the potential to promote equity in diabetes care for racial and ethnic minority populations (2–4). For example, the article by Vrany et al. focuses on strategies to facilitate the use of continuous glucose monitors and virtual care in high-risk people with diabetes, which requires a system-based policy approach (5), and access to and training for these devices (6). Similarly, the findings from Project ECHO Diabetes underscore that tele-education was effective in increasing PCPs’ knowledge, confidence, and skills in diabetes management in rural communities, and could possibly contribute to offset the shortage of endocrinology care in underserved areas.

Another important theme emerging from the articles in this Research Topic is the impact of COVID-19 on the implementation and adoption of telehealth in diabetes care (Addala et al.). The article by Al-Mutairi et al. highlights the effect of telemedicine on glycemic control during the COVID-19 lockdown period and on continuity of care for patients with diabetes during times of crisis. In agreement with recommendations from the diabetes community (7), the authors recommend healthcare systems to develop protocols and comprehensive strategies to manage diabetes during pandemics. They also suggest leveraging virtual consultations and remote patient monitoring (RPM) to provide continued access to care while minimizing the risk of exposure to COVID-19. Although the comparative effectiveness of various e-health interventions remains a matter of debate, the paper by Zhang et al. aims to address this question and suggests that frequent ‘touch points’ using short text messages over 6 months may be superior to standard of care. We anticipate that ongoing studies will uncover key factors for personalizing the type and frequency of e-health interventions to specific subset of people with diabetes, especially in relation with lifestyle interventions. In this context, the article by Dhaver et al. reports that the transition from an in-person to a hybrid model of lifestyle interventions, within the structured Weight Achievement and Intensive Treatment (Why WAIT) program, did not result in any deterioration in the trajectory of weight loss or Hba1c reduction.

Despite the potential benefits of telehealth in diabetes care (8), the articles in this Research Topic also highlight a number of limitations and unsolved questions associated with their implementation. For example, the paper on remote glucose monitoring emphasizes the need for effective data management and patient engagement to ensure the successful use and retention of these technologies (Crossen et al.). This article discusses the feasibility of remote patient monitoring (RPM) for patients and clinicians using a commercially available population health platform. The study shows that remote data-sharing is successful for most participants, with RPM associated with a glycemic management indicator (GMI) change of -0.25% for the entire cohort. RPM was reported to be helpful by 94% of participants, and clinicians’ outreach required a median of 10 minutes per event. RPM has shown promise to enable more individualized and effective care for patients with type 1 diabetes. Further studies and population-wide analytics are needed to focus limited clinical resources on patients who are most in need.

Finally, the COVID-19 pandemic has prompted the rapid adoption of virtual resources for medical trainees and swift changes in the framework of educational platforms (9). The article on post-graduate medical education in the time of COVID-19 underscores the challenges associated with adapting medical education to a remote format, particularly in the context of diabetes care, and highlights the need for new curricula suitable for a telemedicine-based environment (Romeo et al.). The authors also suggest that trainees can potentially spearhead new models of care delivery and innovative approaches to clinical education leveraging telemedicine.

In conclusion, the Research Topic “*Application of Telehealth to Diabetes Care Delivery and Medical Training: Challenges and Opportunities*” features the potential of telehealth technologies to address disparities in diabetes care and promote health equity, while also identifying the challenges associated with their implementation. The articles included in this Research Topic provide valuable insights on the current status and opportunities of telehealth offerings in diabetes care, as well as the need for continued research and innovation to facilitate the adoption of these tools across all demographics. Telehealth has become an important enhancement to traditional in-person medical care, and current efforts to sustain its coverage in the current healthcare landscape are vital to secure continued access for patients. Ultimately, the successful implementation of telehealth in diabetes care will require a collaborative and system-based multidisciplinary approach, involving healthcare providers, patients, medical insurance plans, and technology developers, to ensure that these technologies are effectively integrated into clinical practice and education.

## Author contributions

RM and GR wrote the first draft of the manuscript. All authors contributed to manuscript revision, read, and approved the submitted version.
